# Cysteine Peptidases as Schistosomiasis Vaccines with Inbuilt Adjuvanticity

**DOI:** 10.1371/journal.pone.0085401

**Published:** 2014-01-21

**Authors:** Rashika El Ridi, Hatem Tallima, Sahar Selim, Sheila Donnelly, Sophie Cotton, Bibiana Gonzales Santana, John P. Dalton

**Affiliations:** 1 Zoology Department, Faculty of Science, Cairo University, Cairo, Egypt; 2 National Liver Institute, Menoufiya University, Shebin El-Kom, Menoufiya, Egypt; 3 The i-three Institute, University of Technology Sydney (UTS), Ultimo, Sydney, Australia; 4 Institute of Parasitology, McGill University, Sainte Anne de Bellevue, Canada; 5 School of Biological Sciences, Queen's University Belfast, Belfast, Northern Ireland; Queensland Institute of Medical Research, Australia

## Abstract

Schistosomiasis is caused by several worm species of the genus Schistosoma and afflicts up to 600 million people in 74 tropical and sub-tropical countries in the developing world. Present disease control depends on treatment with the only available drug praziquantel. No vaccine exists despite the intense search for molecular candidates and adjuvant formulations over the last three decades. Cysteine peptidases such as papain and Der p 1 are well known environmental allergens that sensitize the immune system driving potent Th2-responses. Recently, we showed that the administration of active papain to mice induced significant protection (*P*<0.02, 50%) against an experimental challenge infection with *Schistosoma mansoni*. Since schistosomes express and secrete papain-like cysteine peptidases we reasoned that these could be employed as vaccines with inbuilt adjuvanticity to protect against these parasites. Here we demonstrate that sub-cutaneous injection of functionally active *S. mansoni* cathepsin B1 (SmCB1), or a cathepsin L from a related parasite *Fasciola hepatica* (FhCL1), elicits highly significant (*P*<0.0001) protection (up to 73%) against an experimental challenge worm infection. Protection and reduction in worm egg burden were further increased (up to 83%) when the cysteine peptidases were combined with other *S. mansoni* vaccine candidates, glyceraldehyde 3-phosphate dehydrogenase (SG3PDH) and peroxiredoxin (PRX-MAP), without the need to add chemical adjuvants. These studies demonstrate the capacity of helminth cysteine peptidases to behave simultaneously as immunogens and adjuvants, and offer an innovative approach towards developing schistosomiasis vaccines

## Introduction

Schistosomiasis, also known as bilharzia, infects between 391 and 600 million people in 74 developing countries in the tropics and sub-tropics, and close to 800 million, mostly children, are at risk [1]. Disease burden is estimated to exceed 70 million disability-adjusted life-years (DALYS) [2] and leads to remarkably high years lived with disability (YLD) rates [3]. The causative agents are several dioecious (separate sex) trematodes, of the family Schistosomatidae, most notably *Schistosoma mansoni*, *S. haematobium* and *S. japonicum* [1], [2]. Infective aquatic larvae, known as cercariae, invade host skin and develop into schistosomula by exchanging their classical outer membrane for a unique double lipid bilayer as they make their way to the dermal blood vessels [4]. Schistosomula travel via the pulmonary artery to the lungs over 4–6 days before entering the pulmonary capillaries and migrating to the hepatic portal and then to the mesenteric blood vessels. Here, the parasites mature and copulate for their entire life and acquire nutrients from blood to support the production of numerous eggs [5]. There is only one drug available to treat schistosomiasis, praziquantel, and thus the possible emergence of drug resistant parasites could have disastrous public health consequences [1], [2].

Over the last three decades in particular, intense research efforts have been devoted to the discovery of vaccine candidates, a process greatly facilitated by the availability of schistosome proteomic, transcriptomic, and genomic databases [6], [7], [8]. Vaccine trials in mice using various schistosome molecules have demonstrated that significant (*P*<0.05–<0.02) and reproducible reduction of 30–50% in challenge worm burden can be achieved [9]–[13] although no clear consensus has been reached as to the phenotype of immune response required for protection. In our previous studies we showed that during acute natural infection of mice, schistosomula-secreted antigens glyceraldehyde 3-phosphate dehydrogenase (SG3PDH) and peroxiredoxin-multiple antigen peptide (PRX-MAP) elicit predominantly Th1 and Th17 immune responses, characterized by the production of interferon-gamma (IFN-γ) and interleukin (IL)-17, and IgG2a and IgG2b antibodies [14]. We found that immunization of mice with SG3PDH or PRX-MAP alone or emulsified with Freund's, alum, or Allison' adjuvant failed to elicit protection in mice against challenge infection. In contrast, SG3PDH/PRX-MAP combined with the type 2 cytokines, thymic stromal lymphopoietin (TSLP), IL-25, and IL-33 [15], switched the antigen specific response elicited during a challenge infection towards a Th2 phenotype. Circulating antigen-specific antibodies were primarily of the IgG1 isotype and spleen cells stimulated ex vivo, secreted parasite-antigen specific IL-4, IL-5 and IL-13. This alteration in the immune response induced by parasite antigens correlated with high and significant (up to *P*<0.0001) reduction of 62% (TSLP) to 78% (IL-33) in challenge worm burden [16].

Similarly, we discovered that switching the parasite-specific immune response towards a Th2 phenotype by a sub-cutaneous injection of the plant cysteine peptidase papain to mice prior to a challenge infection with *S. mansoni* conferred 50% protection [16]. Papain and other environmental allergens such as ficin, bromelain, and Der p 1 are members of the C-1 peptidases family and their proteolytic activity is believed to be necessary for the adjuvant-like induction of Th2-mediated responses [17]–[20]. Schistosomes express several members of the C-1 peptidases, including cathepsins B (SmCB) and cathepsins L (SmCL) that are known to play critical roles in the digestion of host blood tissues and hemoglobin (SmCB1, SmCL1, SmCL3), in reproduction (SmCL2) and surface tegument biogenesis (SmCB2) [7], [21]–[27]. We reasoned that these schistosome peptidases besides being likely vaccine targets may also, because of their intrinsic proteolytic activity, possess inbuilt adjuvant properties that could enhance their efficacy.

Here we show that sub-cutaneous immunization of outbred mice with functionally active forms of recombinant SmCB1, or a cathepsin L from the related trematode parasite *Fasciola hepatica* (FhCL1) induce highly significant (*P*<0.0001) and reproducible reduction of 50–73% in challenge worm burdens, and in liver and intestinal egg burdens. Blocking of SmCB1 activity with an irreversible inhibitor prior to delivery or use of an enzymatically inactive FhCL1 mutant molecule significantly (*P*<0.0001) reduced, but did not ablate, the protective properties of the peptidase. Higher levels of protection levels (>80%) were attained when either SmCB1 or FhCL1 were delivered in combination with other vaccine candidates, namely SG3PDH/PRX-MAP. These studies demonstrate the capacity of parasite C-1 cysteine peptidases to behave simultaneously as immunogens and adjuvants, and offer an innovative approach towards developing schistosomiasis vaccines.

## Materials and Methods

### Ethics statement

The patient study was approved by the Institution Ethics Review Board of the National Liver Institute, Menoufiya University. All donors of serum samples were adults and provided both verbal and written consent which was also reviewed and approved by the Institution Ethics Review Board and is now stored at Cairo University, Egypt. Studies on mice were approved by the Animal Care and Use Committee of the Theodore Bilharz Research Institute, Giza, Egypt.

### Mice and parasites

Cercariae of an Egyptian strain of *S. mansoni* were obtained from the Schistosome Biological Materials Supply Program, Theodore Bilharz Research Institute (SBSP/TBRI), Giza, Egypt, and used for infection immediately after shedding from *Biomphalaria alexandrina* snails. Outbred, female, six-week-old CD1 mice were raised at SBSP/TBRI, and housed throughout experimentation in the Animal Facility of the Faculty of Science, Cairo University. Every effort was made to minimize animal suffering including change of bedding three times per week, clean, air-conditioned and quiet housing, delicate handling on injection, exposure to infection, and euthanizing, and no extension of experiments beyond seven weeks after infection. All animal experiments were performed following the recommendations of the current edition of the Guide for the Care and Use of Laboratory Animals, Institute of Laboratory Animal Resources, National Research Council, Washington, DC.

### Immunogens

Functionally active *S. mansoni* cathepsin B1 (SmCB1) and *Fasciola hepatica* cathepsin L1 (FhCL1) were expressed in the yeast *Pichia pastoris* GS115 strain using the pPIC9K vector; enzyme activity was assessed by the hydrolysis of 7-amino-4-methyl coumarin (NHMec) from the fluorogenic peptide substrate Z-Phe-Arg-NHMec [28], [29]. The production of the FhCL1 enzymatically inactive variant FhCL1Gly^26^ (Cys^26^ to Gly^26^, mFheCL1) used in this study was described before [29], [30]. Potential N-glycosylation sites where removed from the peptidases cDNAs by mutation so that expressed recombinant did not bear yeast glycans [28]–[31]. The recombinant enzymes were produced by fermentation at 30°C and 250 rpm in 1 liter buffered glycerol-complex medium (BMGY) broth buffered to pH 6.0 into 4 liter baffled flasks until achieving an OD_600_ of 2–6. The cells were centrifuged at 3,000× *g* for 10 min at room temperature and induction initiated by resuspending the pellets in 200 ml BMMY broth and adding 1% of 100% filter–sterilized methanol every 24 h for 3 days. The culture was then centrifuged at 16,000× *g* for 30 min at room temperature and proteases isolated from the supernatant by Ni-NTA affinity chromatography [28], [30]. SmCB was inactivated by incubation for 30 min at room temperature in the presence of 5 µM of the irreversible inhibitor of cysteine peptidases, L-trans-epoxysuccinylleucylamide-(4-guanido)-butane (E-64, Calbiochem), as described [32].

Recombinant *S. mansoni* glyceraldehyde 3-phosphate dehydrogenase (SG3PDH) was prepared and purified to homogeneity, as described [33], and contained <0.06 Endotoxin Units/ml as judged by the Pyrogen Gel-Clot Limulus Amebocyte Lysate test (Bio-Whittaker). 2-Cys peroxiredoxin [34] (H-^104^RKQEISKAYGVFDE EDGNA^122^-OH)-derived peptide, showing lowest homology to the murine counterpart, was synthesized as a tetra branched multiple antigen peptide (MAP) construct and purified at AnaSpec, Inc. (San Jose, California). *Schistosoma mansoni* soluble egg antigen (SEA) was prepared as described [35].

### Mouse immunization and infection

Mice (10–14 per group) were immunized subcutaneously at the base of the tail with 10 or 20 µg SmCB1 or FhCL1 alone or in a mixture, or combined with rSG3PDH and PRX MAP (10 µg/mouse), twice (unless otherwise stated) at 3-week interval. Two (unless otherwise stated) weeks after the last injection, unimmunized and immunized mice were infected percutaneously via whole body exposure to 120±5 (Experiments 1, 2, 3, 6 and 7) or 140±5 (Experiments 4 and 5) viable cercariae of *S. mansoni*.

### Recovery of lung-stage schistosomula

For each experiment, lung-stage schistosomula were recovered from 2–3 mice per group on day 6 post infection, as described [36], and counted on an individual mouse basis.

### Spleen cell cultures and cytokine assays

Spleen cells (SC) were harvested from 2–3 mice per group per experiment on day 6 after infection with *S. mansoni* cercariae, and resuspended on an individual mouse basis in RPMI-1640 medium supplemented with 200 U/ml penicillin, 200 µg/ml streptomycin, 25 mM HEPES, 50 ng/ml amphotericin, 20 µg/ml polymyxin B (Sigma) as an inhibitor of any residual lipopolysaccharide contamination of recombinant antigens, 5×10^−5^ M 2-mercaptoethanol, and 10% fetal calf serum (culture medium) [14], [16]. Splenocytes were cultured at a concentration of 1×10^6^ cells/200 µl culture medium/well in duplicate wells of 96 round-bottomed well plates (Corning Costar), stimulated with 0 or 5 µg/ml membrane filter (0.45 µm)-sterilized immunogen, and maintained at 37°C in a humidified atmosphere containing 3.0% CO_2_. At 48 and 72 h of incubation, cultured SC were thawed and frozen for release of intracellular cytokines, and supernatants stored at −76°C until assayed by capture enzyme-linked immunosorbent assay (ELISA) for concentrations of IL-4, IL-5, IL-17A, and IFN-γ (ELISA MAX™ Set, BioLegend) and IL-13 (DuoSet ELISA Development System, R&D Systems Europe) following the manufacturer's instructions.

### Mouse humoral responses

Sera were obtained from unimmunized and immunized mice 6 days following infection with cercariae of *S. mansoni*, and individually assessed by ELISA for humoral antibody titer reactivity to the immunogens (250 ng per well). For each experiment, antibody isotypes were determined using rat alkaline phosphatase-conjugated monoclonal antibodies to mouse IgG subclasses (Pharmingen) with mouse sera diluted 1∶200, and biotin-labeled monoclonal antibody to mouse IgM, IgA, and IgE (BioLegend) with sera diluted 1∶50, as described [16].

### Worm and worm egg recovery

Worm burden and liver and intestine worm egg load in individual mice (7 to 11 per group) were evaluated 40–49 days after challenge infection, as described [37]. Mean values ± SD for each group were calculated. Percent change was evaluated by the formula: % change = mean number in infected controls−mean number in infected, immunized mice/mean number in infected controls×100.

### Human serum samples and antibody isotype reactivity

Serum samples were obtained from 50 patients, 18–20 year-old, attending the outpatient clinic of the National Liver Institute, Menoufiya University, Egypt, and requesting parasite infection diagnosis. For each donor, two microscopic slides of stool samples were examined on 3 consecutive days by the Kato-Katz method, as described [33]. Informed consent was obtained from each patient with confirmed schistosomiasis (100–400 eggs per gram stool). Sera from parasite-free and *S. mansoni*-infected donors were tested by ELISA for IgM, IgG1, IgG2 (serum diluted 1∶250), IgG4, IgA1/A2, and IgE (serum diluted 1∶25) antibody binding to 250 ng/well recombinant SmCB1, FhCL1, SG3PDH, or 1 µg/well SEA. Alkaline phosphatase- or biotin-labeled monoclonal antibodies to human immunoglobulin isotypes were obtained from BD Biosciences (Franklin Lakes, New Jersey), and used at 1∶1000, and 1∶500 dilution, respectively. Alkaline phosphatase-labeled streptavidin was purchased from Promega (Madison, Wisconsin), and used at 1∶3000 dilution.

### Data analysis and statistics

All values were tested for normality. Student's unpaired 2-tailed *t*-test, Mann–Whitney, and ANOVA tests were used to analyze the statistical significance of differences between experimental and control values and considered significant at *P*<0.05.

## Results

### Vaccination against murine schistosomiasis with C-1 cysteine peptidases

**Figure 1:**
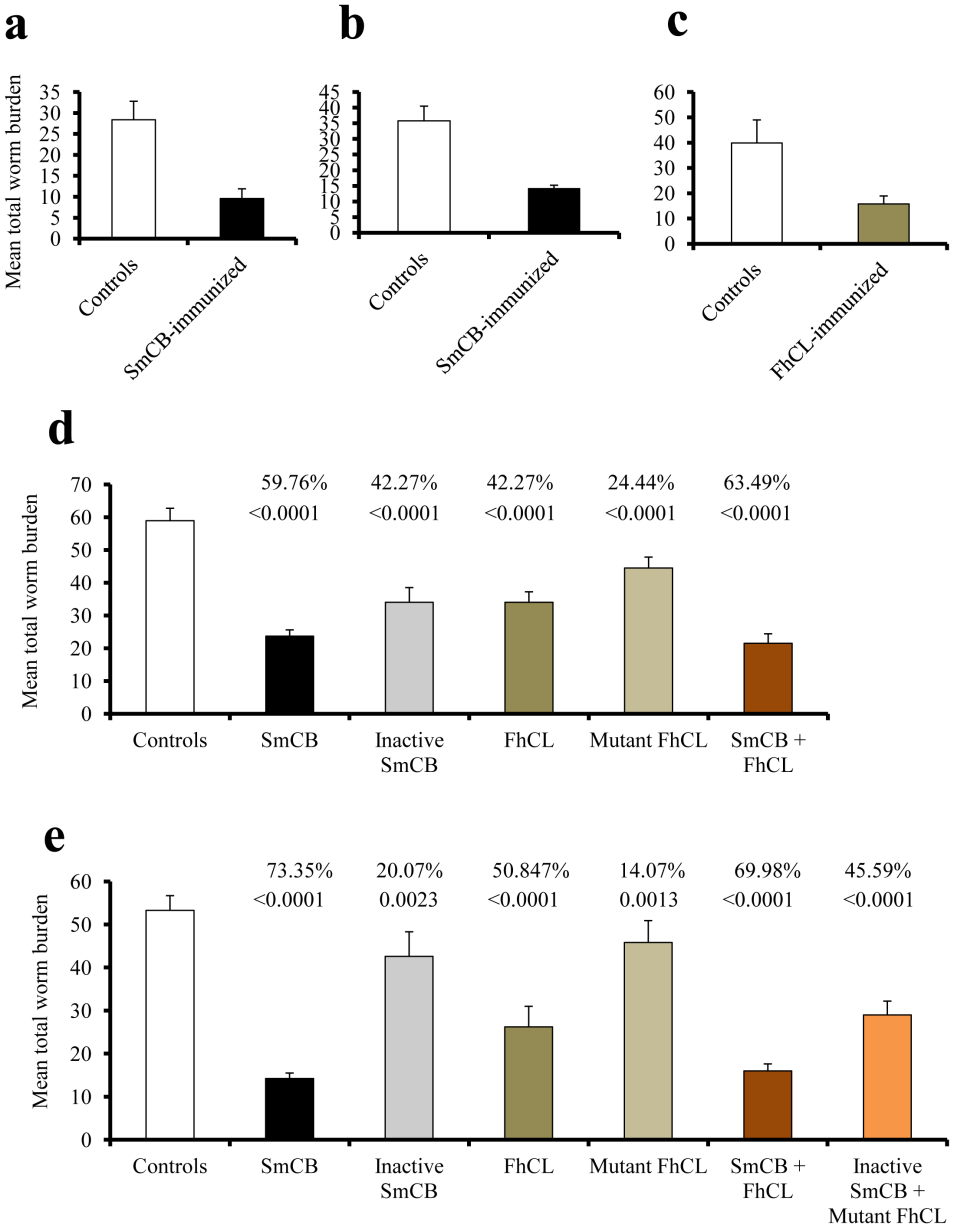
Cysteine peptidases vaccine potential. Mice were immunized 1x (a) or 2x (b–e) with 10 (a–d) or 20 (e) µg active (a–e) or inactive (d,e) cysteine peptidase/mouse/injection, alone (a–e) or in a mixture (d,e), and exposed 15 days later to 120 (a–c) or 140 (d,e) *S. mansoni* cercariae. Columns represent mean worm burden in 7–14 mice/group, and vertical bars denote SD about the mean. *P* values and percent reduction in worm burden as compared to control mice are shown above the columns of the test groups. Differences in mean worm burden between mice immunized with active and inactive cysteine peptidases (d,e) are highly significant (*P*<0.0001).

The major C-1 cysteine peptidase of the blood fluke *S. mansoni*, SmCB1, was expressed as a functionally-active recombinant enzyme using the yeast *Pichia pastoris* as the surrogate host. The enzyme was purified to homogeneity by Nickel-affinity chromatography and was injected (10 µg) subcutaneously into mice (number = 10–14) either once, or twice with a two-week interval. Two weeks later the mice were infected with *S. mansoni* cercariae and worm burden assessed 40–49 days post infection. Administration of parasite C-1 peptidase induced highly significant (*P*<0.0001) reduction (>60%), in total (Figures 1a,b), and male and female challenge worm burden, accompanied with significant (*P*<0.005) decrease in worm egg counts in liver, but not small intestine (Table 1).

**Table 1:** Protective effects of immunization with C-1 peptidases SmCB1 and FhCL1 alone and in combination with SG3PDH/PRX-MAP on male and female worm burdens and parasitological parameters.

Experiment	Vaccine formulation	% Reduction in Worm burden*			% Reduction in eggs in liver	% Reduction in eggs in small intestine
		Total	Males	Females		
**1. Single immunization**	SmCB1	60.05 (<0.0001)	60.29 (<0.0001)	59.57 (<0.0001)	26.63 (<0.0041)	26.01 (<0.018)
	SmCB1&SG3PDH/PRX-MAP	83.79 (<0.0001)^a^	88.23 (<0.0001)	82.44 (<0.0001)	54.68 (<0.0001)	37.58 (0.0006)
**2. Two immunizations**	SmCB1	66.19 (<0.0001)	65.88 (<0.0001)	66.55 (<0.0001)	51.30 (<0.0007)	24.71 (NS)
	SmCB1&SG3PDH/PRX-MAP	75.00 (<0.0001)	74.1 (<0.0001)	75.60 (<0.0001)	58.44 (0.0002)	41.24 (0.0096)
**3. Two immunizations**	FhCL1	60.40 (<0.0001)	60.67 (<0.0001)	59.89 (<0.0001)	34.99 (<0.0007)	24.05 (NS)
	FhCL1&SG3PDH/PRX-MAP	73.43 (<0.0001)	73.78 (<0.0001)	72.91 (<0.0001)	60.10 (<0.0001)	49.38 (<0.0001)
**4. Single immunization**	SmCB1&FhCL1&SG3PDH/PRX-MAP	56.45 (<0.0001)	52.84 (<0.0001)	61.73 (<0.0001)	58.84 (<0.0001)	65.0 (<0.0001)
**5. Two immunizations**	SmCB1&FhCL1&SG3PDH/PRX-MAP	66.39 (<0.0001)	66.66 (<0.0001)	65.97 (<0.0001)	65.97 (<0.0001)	69.32 (<0.0001)

Mice (10–12/group) were immunized with SmCB1 or FhCL1 combined with rSG3PDH/PRX-MAP and challenged 2 (panels 1–3) or 3 (panels 4 and 5) weeks after the last immunization.

* Percent reduction was evaluated by the formula: % reduction = mean number in infected controls−mean number in infected, immunized mice/mean number in infected controls×100.

a *P* values as calculated by Student 2-tailed *t*-test. NS = not significant.

In confirmatory experiments, using two immunizations of different doses of SmCB1, 10 µg (Fig. 1d) or 20 µg (Figure 1e), injection of active peptidase induced a protection level of 60% and 73%, respectively. Furthermore, to determine whether the protective properties of the SmCB1 were dependent on its proteolytic activity we performed immunizations with SmCB1 that had been incubated with the irreversible covalent C-1 peptidase inhibitor E- 64 just prior to injection into mice; this treatment significantly (*P*<0.0001) reduced the vaccine efficacy to 42% (Fig. 1d) and 21% (Figure 1e), respectively, although these proved to be still statistically significant (*P*<0.0001; *P* = 0.0023) when compared to non-vaccinated infected mice.

To verify that this immune protection is a generic property of C-1 peptidases we also tested a functionally-active cathepsin L peptidase (FhCL1) derived from another trematode, the liver fluke *F. hepatica*. Subcutaneous injection of this peptidase induced highly significant (*P*<0.0001) levels of protection of 60% (Figure 1c) and decrease in liver but not small intestine worm egg load (Table 1). The data were confirmed in two subsequent experiments showing protection of 42% and 51% (Figures 1d,e) against a challenge *S. mansoni* infection, achieved with 10 µg or 20 µg doses, respectively. A non-functionally active but structurally sound mutant of FhCL1 (mFhCL1) [29], [30] induced protection of only 24% (*P*<0.0001) and 14% (*P* = 0.0013) (Figures 1d,e). Differences in mean worm burden between mice immunized with active and inactive FhCl (Figures 1d,e) are highly significant (*P*<0.0001), indicating that protection with this peptidase was also associated with its cysteine peptidase activity.

A mixture of SmCB1 and FhCL1 (10 µg each per injection) induced 63% and 70%, reduction in worm burden, respectively (Figures 1d,e), which was not significantly different to using SmCB1 alone, but elicited highly significant (*P*<0.005) reduction in worm egg load in liver and small intestine (data not shown). Accordingly, the collective data show that subcutaneous delivery of functionally active worm C-1 peptidases alone, or as mixtures, can induce consistent and high levels of protection in mice against a challenge infection with cercariae of *S. mansoni*.

### Cysteine peptidases immunogenicity

**Figure 2:**
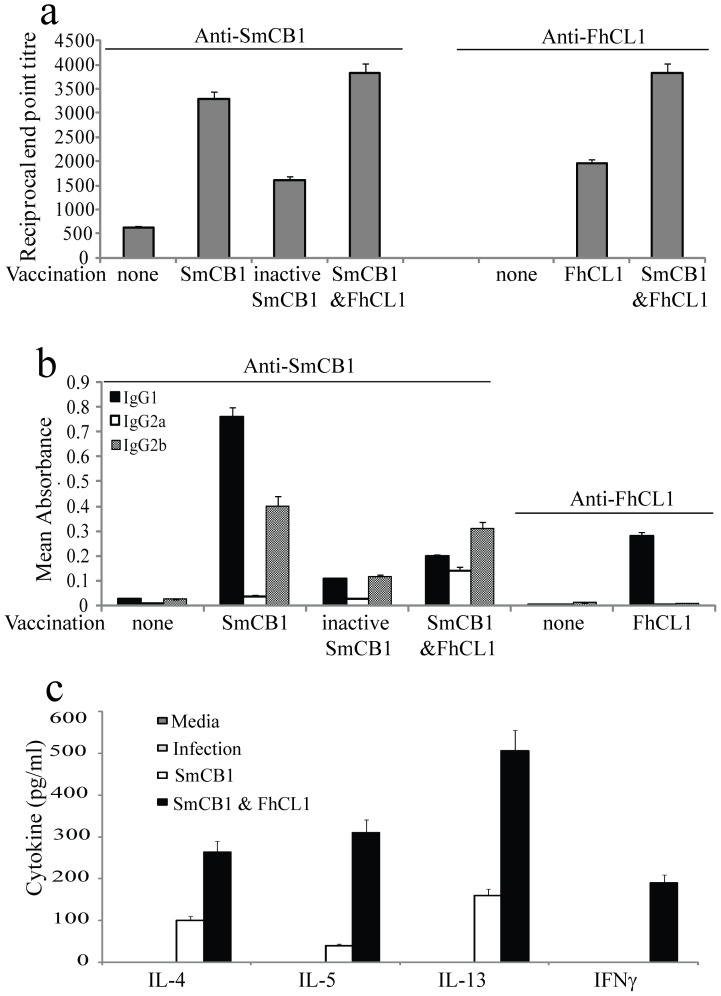
Effects of immunization with SmCB or FhCL without adjuvant on immune responses, assessed 6 days post infection. (a) Titres of peptidase specific IgG were measured in the sera of immunized mice by ELISA. The data shown is representative of 3 independent experiments and displays the inverse of the end point serum dilution. (b) The isotype of peptidase specific antibodies in the sera of mice was determined by ELISA. The data shown is representative of 5 independent experiments. Each column represents the delta mean absorbance ± the standard error around the mean (SD). (c) Spleens were harvested from mice that were infected following immunization with SmCB1 or a combination of SmCB1 and FhCL1. The quantity of cytokine (IL-4, IL-5, IL-13 and IFN-γ) released into culture media by spleen cells stimulated with SmCB1 in vitro was measured by ELISA. Supernatants from cells incubated in media only served as negative controls. Each column represents the mean ± SD.

Mice infected with *S. mansoni* produced low levels of SmCB-specific IgG six days after infection, the titre of which was significantly enhanced in mice pre-immunized with SmCB1 (Figure 2a). In contrast, the increase in peptidase-specific IgG in mice immunized with inactivated SmCB1 was not significant, perhaps reflecting the reduced level of protection seen in these animals compared to vaccination with SmCB1. The titre of SmCB1 antibody observed in infected mice, pre-immunized with a combination of SmCB1 and FhCL1 was similar to that induced by SmCB1 alone, mirroring the protective efficacy (Fig. 2a). Infected mice that had been vaccinated with FhCL1 produced a similar titre of peptidase-specific IgG to that of mice immunized with inactivated SmCB1 (Figure 2a), which again correlates with the similar levels of protection observed in these particular groups. These peptidase-specific antibodies were of the IgG1 and IgG2b isotypes (Figure 2b), indicating bias towards a Th2 type immune response [38], despite the larval antigens induction of predominant Th1 and Th17 immune responses at 6 days after infection [14].

In support of the antibody data, mice infected with *S. mansoni* that had been pre-immunized with *S. mansoni* peptidase SmCB1, developed a Th2-type antigen specific immune response. Spleen cells obtained from these mice showed increased secretion of Th2-associated cytokines IL-4, IL-5, IL-13 but not Th1 type cytokine IFN-γ in response to stimulation with SmCB1 in vitro (Figure 2c). The amount of cytokines secreted from spleen cells isolated from mice vaccinated with either inactive SmCB1 or FhCL1, the least protective preparations, was below the sensitivity threshold of the cytokine assay used, and thus no different to spleen cells harvested from non-immunized, infected mice (data not shown). In contrast, the mixture of both FhCL1 and SmCB1, induced a mix of Th1 and Th2 type peptidase-specific cytokines, although the overall levels of IL-4, IL-5 and IL-13 are higher than IFN-γ, indicating a bias towards a Th2 phenotype (Figure 2c).

### Parasite cysteine peptidases act as adjuvants for schistosome vaccine candidates

We investigated whether C-1 peptidases could act as adjuvants in combination with other schistosome vaccine candidates (Table 1). We chose the antigen mix of SG3PDH and PRX-MAP that was previously shown to induce less than 10% protection in mice when delivered alone or in combination with Freund's, Allison's or alum adjuvant, but elicited 62–78% reduction in challenge worm burden and worm eggs when combined with Th-2 inducing cytokines [16]. In the first experiment, a single immunization of SmCB1 alone induced a protection level of 60% (*P*<0.0001), while the addition of the SG3PDH/PRX-MAP antigens increased protection to 84% (*P*<0.0001). In a second experiment, two immunizations with SmCB1 resulted in a protection level of 66% (*P*<0.0001) while inclusion of the SG3PDH/PRX-MAP antigens elicited a protection level of 75% (*P*<0.0001) (Table 1). Addition of the SG3PDH/PRX-MAP antigens to FhCL1 peptidase also increased the protection levels, in this case from 60% to 73% (Table 1). In all trials, highly significant (*P* = 0.0096–<0.0001) reductions in worm egg burden in liver and small intestine were achieved, yet with values lower than for the worm burden (Table 1).

**Figure 3:**
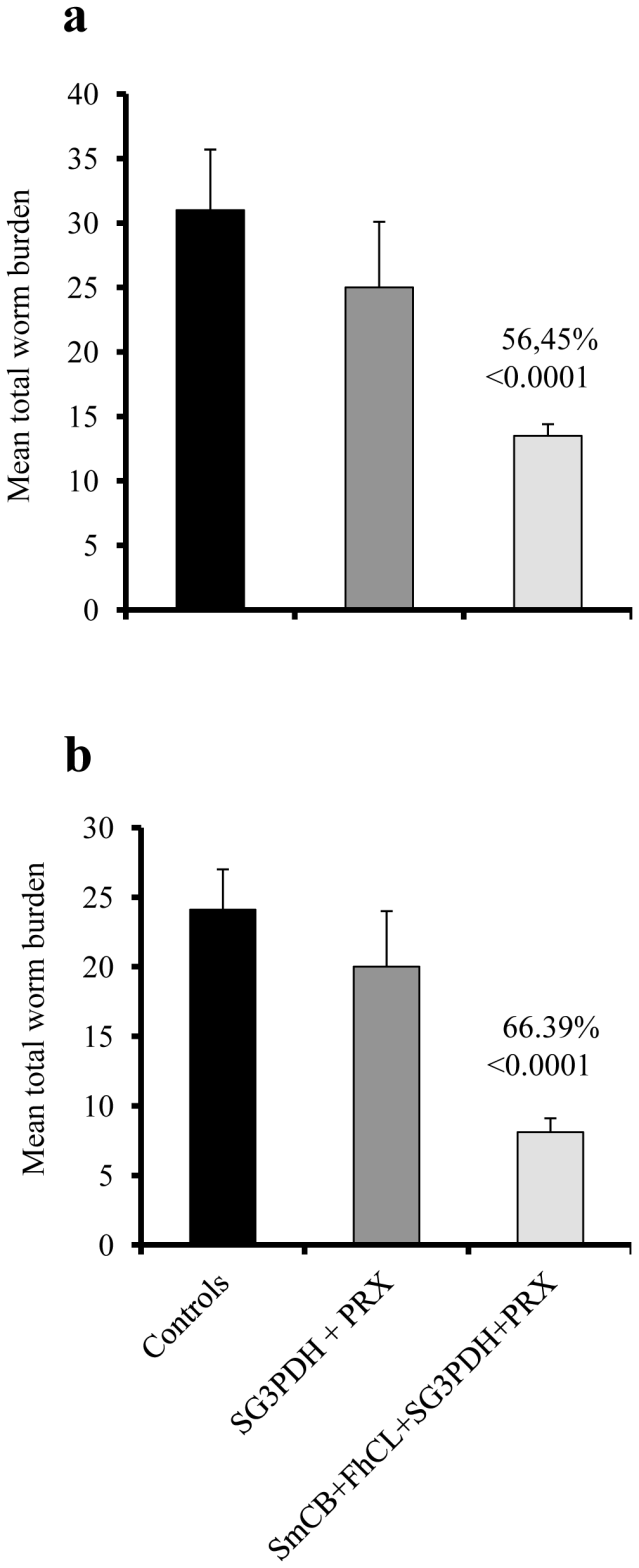
Adjuvant effect of SmCB and FhCL mixture. Mice were immunized 1x (a) or 2x (b) with rSG3PDH+PRX-MAP alone or combined with SmCB+FhCL, and challenged 3 weeks later with 120 cercariae of *S. mansoni*. Columns represent mean worm burden in 8–12 mice/group, and vertical bars denote SD about the mean. *P* values and percent reduction in worm burden and as compared to control mice are shown above the columns of the test groups.

In two experiments, mice were immunized once (Figure 3a) or twice (Figure 3b) with a mixture of functionally active SmCB1 and FhCL1 in combination with the SG3PDH/PRX-MAP antigens. Protection levels were not boosted beyond using C-1 peptidase alone (Figure 3, Table 1) but we observed very high reductions (*P*<0.0001) of 58–69% in the number of eggs recovered from the liver and small intestine of the challenged mice (Table 1).

To obtain information regarding the stage of development at which the C-1 peptidases vaccine may operate, a number of mice from each group were sacrificed 6 days following the challenge infection and the lungs examined for migrating immature parasites. No attrition of challenge worms was apparent at this time as the number of schistosomula recovered from the lungs did not differ between unimmunized and immunized mice (Table 2). Interestingly, examination of the lungs at 6 days after infection indicated that the combined vaccine of SmCB1 and SG3PDH/PRX-MAP elicited significant protection against the early migratory stages of the parasite (Table 2).

**Table 2:** Recovery of lung-stage schistosomules from unimmunized and immunized mice 6 days after the challenge infection.

Vaccine formulation	No. of mice tested	No. of larvae/mouse ± SD	*P* value
**PBS (controls)**	12	16.1±3.9	-
**SmCB1**	10	15.1±4.7	NS
**FhCL1**	8	15.1±3.1	NS
**SmCB1+FhCL1**	8	13.2±3.6	NS
**SmCB1+SG3PDH/PRX MAP**	6	7.5±3.4	0.0003*

For each experiment, lung-stage larvae were recovered from 2–3 mice per group, and counted on an individual mouse basis. Mean number ± SD around the mean of larvae retrieved from 2–6 repeat groups is shown. NS = not significant compared to PBS controls, as assessed using the Student 2-tailed *t*-test.

* Immunization of mice with SG3PDH or PRX-MAP alone or emulsified with Freund's, alum, or Allison' adjuvant failed to elicit changes in number of lung-stage larvae recovered from immunized mice compared to adjuvant controls (data not shown).

### Human antibody isotype responses

**Figure 4:**
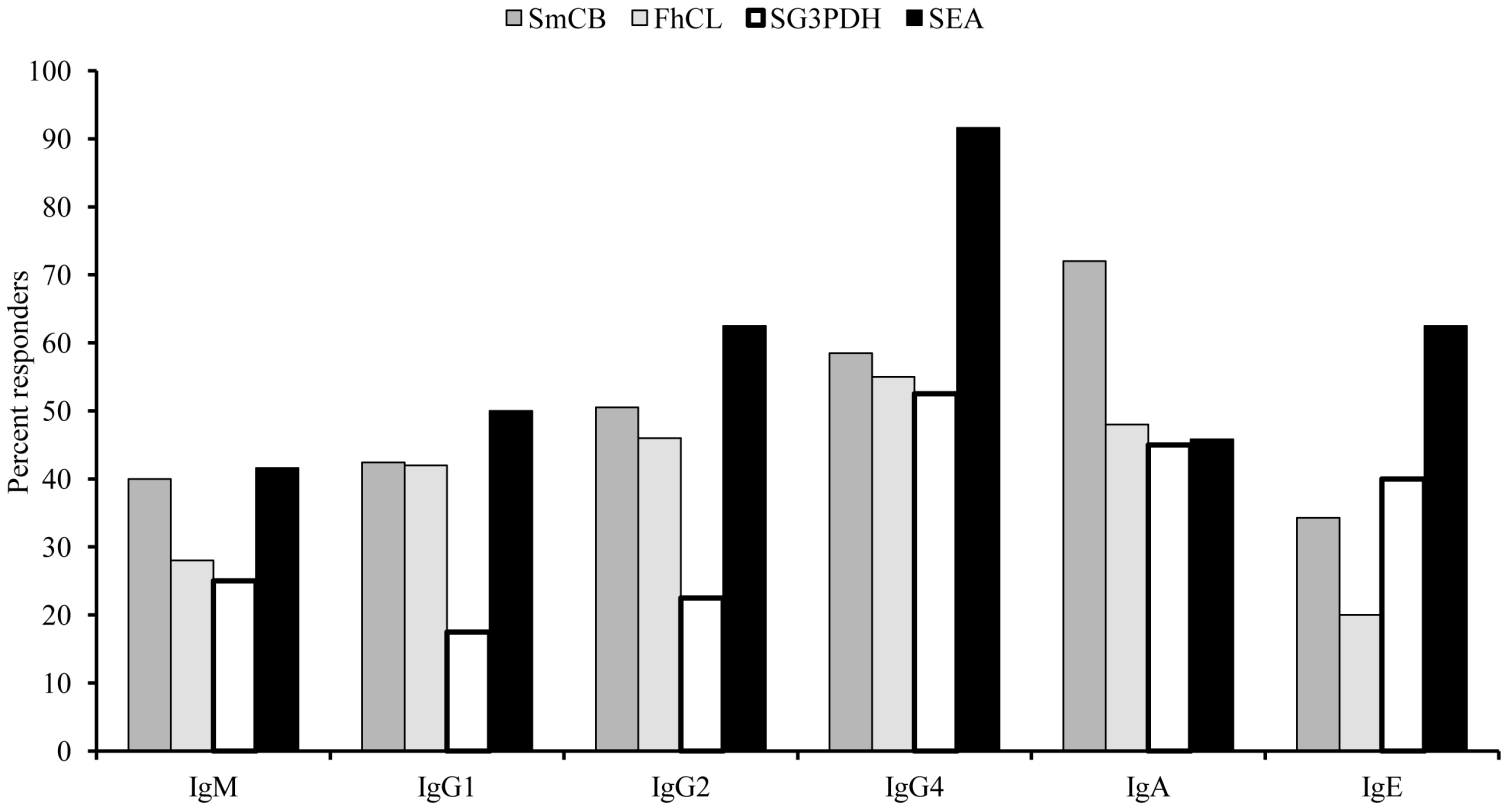
Human antibody isotype responses. A total of 50 patients (18–20 year-old) with confirmed schistosomiasis (100–400 eggs/gram stool) were tested for serum antibody IgM, IgG1, IgG2 (sera diluted 1∶250), IgG4, IgA1/A2, and IgE (sera diluted 1∶25) reactivity to recombinant SmCB, FhCL, or SG3PDH (250 ng/well), or SEA (1 µg/well) in duplicate wells. Patients with mean absorbance values higher than the mean absorbance values of 14 sex- and age-matched, parasite-free, control donors are considered responders.

In order to examine whether helminth cysteine peptidases induce allergenic reactions we examined the humoral responses to SmCB1 and FhCL1 in parallel toSG3PDH and SEA, during natural infection in humans. Antibody analysis of sera obtained from 50 patients with patent schistosomiasis revealed that antibodies binding to SmCB1, FhCL, or rSG3PDH were essentially of the IgG2, IgG4, and IgA isotypes with little IgM, IgG1, and IgE. Conversely, antibodies binding to SEA were predominantly of the IgM, IgG2, IgG4 and IgE isotype (Figure 4).

## Discussion

Our previous studies have shown that immunization with larval excretory-secretory products (ESP) in a recombinant or multiple antigen peptide (MAP) construct in combination with the cysteine protease papain or the type 2 cytokine, TSLP, IL-25, or IL-33 consistently induced highly significant (*P*<0.0001) reduction of 60–78% in challenge *S. mansoni* worm burden and worm egg load in liver and small intestine of outbred mice [16]. Since helminth cysteine peptidases are both ESP [21]–[23] and potentially type 2 immune responses-inducing [17], [20], it was reasonable to assess the adjuvant-free vaccine potential of the cysteine peptidases SmCB, and FhCl, which shows 60% homology with the *S. mansoni* counterpart.

We here demonstrate that the sub-cutaneous administration of functionally active *S. mansoni* C-1 peptidase, SmCB1, to mice elicits consistent and remarkably high levels protection against a challenge infection of the *S. mansoni* parasite; we observed >60% reduction in worm burden and a decrease in worm eggs trapped in liver. The induction of high-level protection was related to the native proteolytic activity of the peptidase as inactivation of the enzyme with an inhibitor significantly (*P*<0.0001) reduced, although did not ablate, its protective activity. Moreover, the functional C-1 peptidase derived from *F. hepatica*, FhCL1, was also capable of inducing high-level anti-schistosome protective immune responses, while its inactive variant induced significantly (*P*<0.0001) lower protection levels. Therefore, these data support our suggestion that helminth C-1 peptidases can induce protection against *S. mansoni* infection in mice.

Injections of SmCB1 or FhCL1 peptidases significantly increased the titres of antigen-specific antibody responses within 6 days after challenge infection compared to mice receiving a schistosome infection alone. The antibodies detected in the peptidase-immunized mice were primarily of the IgG1 and IgG2b isotype (no IgE was detected), demonstrating that the injection of the peptidases boosted early adaptive immune responses with CD4^+^ T cell help. Furthermore, peptidase-specific cytokine secretion, particularly the Th2 cytokines IL-4, IL-5, IL-13, was also observed in the immunized mice at 6 days after infection but not in the non-immunized mice. It has been previously shown that the subcutaneous injection of several C-1 allergens, such as papain and bromelain, produce Th2 adjuvant activity, enhancing Th2-type responses to bystander antigens such as ovalbumin in mice [18]–[20], [39]–[41]. We suggest, therefore, that the protective immune responses generated by peptidase injection likely result from skewing of the immune response in a way that does not support the development of schistosome parasites. In addition, the induction of peptidase-specific immune responses, particularly antibodies, likely bind and prevent the action of these essential enzymes. This latter idea may explain why inactive SmCB1 peptidases also elicited a low but significant level of protection. The mutant FhCL1 induced a minimal level of protection which may be accounted for by the induction of antibodies that cross-react with schistosome cathepsin L peptidases.

Despite observing specific peptidase-induced immune responses in the first week of the challenge infection these did not cause a reduction in the number of lung-stage schistosomula implying that parasite attrition occurs sometime after their migration from the lung, perhaps in the liver [42], or after settlement of the parasites in the mesenteric veins. This is interesting in the context of previous studies suggesting that schistosomes not only promote the differentiation of CD4^+^ T cells but also depend on their activity for their successful maturation in the mesenteric veins and subsequent egg production [43]. The pre-patent immune responses to schistosomes is generally considered to be dominated by Th1-responses [44], although recently de Oliveira Fraga et al. [45], [46] showed that female and males worms also induce antigen-specific Th2 responses. Upsetting the fine balance between the Th1 and Th2 responses in pre-patent infection in either direction, may be sufficient to achieve protection. Consistent and high-level protection is also observed when mice are exposed to irradiated-attenuated cercariae but, in contrast to our observations with SmCB1 and FhCL1, this is effective against the parasites as they migrate into the lungs and is mediated by Th1-driven responses [42], [47].

In our previous studies we showed that we could induce highly significant (*P*<0.0001) reduction (60–78%) in the worm burdens and worm egg load in liver and small intestine in mice challenged with *S. mansoni* when the larval excretory-secretory antigens SG3PDH/PRX-MAP were administered subcutaneously with papain. Antibody and cytokine analysis confirmed that papain was facilitating a bystander Th2-like adjuvant effect on SG3PDH/PRX-MAP. Furthermore, the levels of protection obtained were similar to those observed when SG3PDH/PRX-MAP was delivered in combination with Th2-associated cytokines, TSLP, IL-25, or IL-33 [16]. Here we showed that when SG3PDH/PRX-MAP was combined with SmCB1, or FhCL1, we could achieve very high levels of protection, up to 83%. The combination of SG3PDH/PRX-MAP and parasite C-1 peptidases also exhibited a blocking effect on lung-stage schistosomes, consistent with our earlier findings [16] and elicited a profound reduction in eggs trapped in the liver and intestinal tissues of the mice. These data demonstrate the potential for other schistosome antigens to act in synergy with the C-1 peptidases and prompt future trials using other protective antigens that are known to target the early migratory stages, such as IRV5 and calpain [9]–[13].

It is interesting to note that immunizations with SmCB1 and FhCL1 alone or in combination with rSG3PDH/PRX-MAP did not induce a considerable decrease in worm egg counts in the small intestine when assessed at or before six weeks after infection (data not shown), in support of earlier findings documenting a correlation of Th2 dominant responses with increased production of schistosome eggs [48], [49]. However, we observed highly significant (*P*<0.002) reduction in egg load at seven weeks after infection, in contrast to vaccination with radiation-attenuated cercariae, which induced a transient decrease in *S. mansoni* fecundity that was restored to normal at eight weeks post infection [50].

Highest (*P*<0.0001) reduction in challenge worm egg counts in liver and small intestine was consistently recorded when SG3PDH/PRX-MAP was combined with SmCB1 and FhCL1, which likely cross-reacts with SmCL1 enzymes that possess a reproduction-related function [24], [29], [51]. Therefore, in the future is may be worthwhile examining the effect of immunization with functional C-1 peptidase of schistosomes that are believed to be involved in a variety of functions including reproduction and tegument biogenesis [21]–[27]. It has previously been shown that treatment of *S. mansoni*-infected mice with an inhibitor of cysteine peptidases led to reduction in parasite burden and pathology [52].

Using active C-1 peptidases derived from schistosomes may offer an innovative approach to vaccine and/or adjuvant design, especially that SmCB and FhCL failed to induce generation of specific IgE after one or two immunizations in mice. In contrast to the findings of Chappell et al. [53], we did not observe adverse reactions in any immunized mouse at 6 or 7 weeks after infection. Additionally, serum antibodies of humans infected with *S, mansoni* binding to SmCB1 and FhCLwere predominantly of the IgG and IgA isotype, in support of previous studies [53], [54], providing a barrier to potential adverse sensitization. Data shown in Figure 4 revealed that the number of patients with cysteine peptidase-specific IgE did not differ from the number of patients responding with IgE antibodies to SG3PDH [33]. Additionally, the cysteine peptidase-specific IgE antibody levels were low corroborating the findings of de Oliveira Fraga et al [46]. It is important to note that the data shown in Figure 4 support and extend the findings documenting the multiple specificities of schistosome antigens-specific IgE, precluding dimerization of IgE/IgE receptors on the surface of basophils or mast cells, and consequent allergic reactions.

In conclusion, we report a highly effective vaccine using functionally active cysteine peptidases in outbred mice and where immunological correlates of resistance is associated with Th2 responses consistent with that found in humans for all three species of schistosomes [55]–[58].
